# High temperature hyperthermia treatment for canines exhibiting superficial tumors: A report of three cases

**DOI:** 10.3892/ol.2014.2496

**Published:** 2014-09-03

**Authors:** HIDEFUMI TAKAGI, KAZUO AZUMA, TOMOHIRO OSAKI, NORIHIKO ITOH, SHINICHI NAKAZUMI, YASUHO TAURA, YOSHIHARU OKAMOTO

**Affiliations:** 1 Department of Clinical Veterinary Science, The United Graduate School of Veterinary Science, Yamaguchi University, Yamaguchi 753-8515, Japan; 2 Takagi Animal Clinic, Saijo, Ehime 793-0035, Japan; 3 Department of Veterinary Clinical Science, School of Veterinary Medicine, Faculty of Agriculture, Tottori University, Tottori 680-8533, Japan; 4 AdMeTech Co., Ltd, Matsuyama 790-0054, Japan

**Keywords:** high temperature hyperthermia, canine, spontaneous tumors

## Abstract

High temperature hyperthermia (HTH) treatment has previously been demonstrated to suppress tumor growth in a tumor-bearing rat model. In the present study, the effects of HTH therapy for the treatment of spontaneous tumors in canines was evaluated. In case 1, an 18-year-old female Papillon presented with a right forelimb rhabdomyosarcoma. Case 2 was a 13-year-old male English Cocker Spaniel with a right external auditory canal ceruminous adenocarcinoma and case 3 was a 14-year-old male Golden Retriever that exhibited a perianal gland adenocarcinoma, which surrounded the anus. HTH treatment was performed in all three cases for 10 min at 45–65°C with or without the inhalation of isoflurane. In case 1, the tumor disappeared four weeks following HTH treatment. In case 2, the tumor volume had decreased by day 21, and in case 3, HTH was performed three times and the tumor disappeared following the third procedure. HTH is considered to be a simple procedure with no severe side effects. Consequently, this treatment modality is hypothesized to become a useful alternative therapy for superficial tumors in companion animals.

## Introduction

The life span of companion animals has been prolonged by the advent of routine vaccinations, improved nutrition and living environments, and advances in veterinary medicine. As a result, the incidence of aging-associated illnesses has increased in the companion animal population. Specifically, cancer is considered to be a significant issue. As in human medicine, there are three major modalities for cancer treatment within veterinary medicine; surgery, chemotherapy and radiation therapy. However, it is difficult to treat all of the affected animals with these types of therapy due to the cost and the limited number of facilities available. Therefore, the development of novel treatment strategies is required.

Conventional hyperthermia has long been established as a treatment for cancer, particularly for superficially located tumors (1). Conventional hyperthermia is performed alone or as an adjunct to radio- or chemotherapy (2–5) and has previously been adopted to treat spontaneous tumors in veterinary medicine (6–9). Various studies have focused on two common strategies; conventional hyperthermia at mild temperatures (range, 42–45°C) (1,10,11) and ablation therapy at high temperatures (>70°C) (12). Our previous study demonstrated that high temperature hyperthermia (HTH) treatment ranging between 60 and 70°C suppressed glioma tumor growth and induced necrosis and apoptosis in a rat model (13). In the present study, the efficacy of HTH therapy in the treatment of spontaneous tumors in canines is evaluated.

## Case report

### Case 1

**Figure 1:**
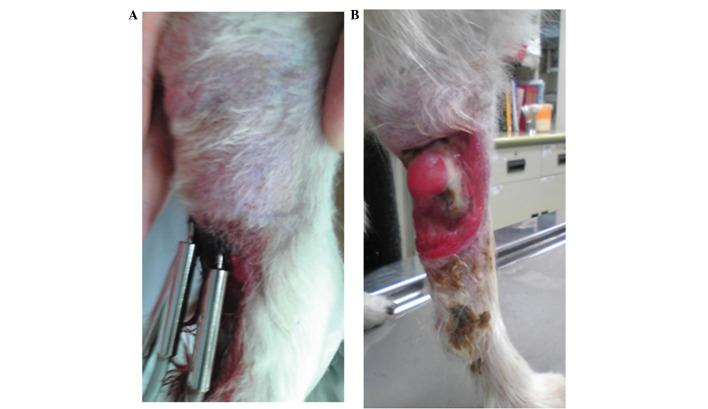
Gross appearance of case 1. (A) A rhabdomyosarcoma of the right forelimb. (B) On day 21, the tumor volume had decreased compared with the volume that was observed on day 0.

An 18-year-old female Papillon (weight, 3.2 kg) was referred to the Yamaguchi University Veterinary Teaching Hospital (Yamaguchi, Japan) in April, 2010 for evaluation of a right forelimb tumor (Fig. 1A). Surgical excision of the tumor had been performed twice previously, however, the tumor had recurred. Histological analysis revealed that the tumor was a rhabdomyosarcoma. On initial examination, the caudal right forelimb was covered by the tumor and the animal was incapacitated in the affected limb. The risk of recurrence and the treatment options were explained to the owners, which included surgery, radiation therapy and chemotherapy. Complete surgical excision was considered to be too complex, as the tumor border was unclear. HTH experimental therapy was recommended and the animal was enrolled in the clinical trial, with the owners’ written informed consent. A tissue ablation device for veterinary medicine (AMTC 200; AdMeTech Co., Ltd., Ehime, Japan) was used to administer the HTH treatment. On day 0, HTH therapy was performed with no anesthesia or sedation. Three needles of the device were inserted into the tumor tissue at 6-mm intervals and the HTH therapy was performed for 10 min at 65°C. On day 21, the tumor volume had decreased from that which was observed on day 0, and the subject had regained improved function of the limb (Fig. 1B). Following four weeks of HTH therapy, the tumor disappeared.

### Case 2

**Figure 2:**
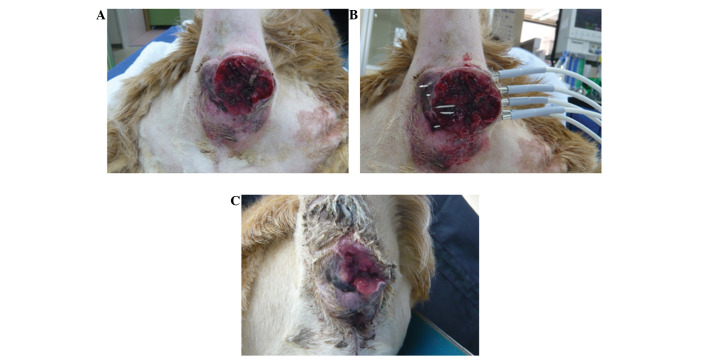
Gross tumor appearance in case 2. (A) The tumor surrounding the anus was later diagnosed as a perianal gland adenocarcinoma. (B) High temperature hyperthermia (HTH) treatment was performed under general anesthesia. Five needles of the ablation device were inserted into the tumor, and HTH therapy was performed for 10 min at 65°C. (C) On day 21, the tumor volume had decreased compared with the volume that was observed on day 0.

A 14-year-old male Golden Retriever (weight, 32.7 kg) was referred to the Takagi Animal Clinic (Saijo, Japan) in February, 2011 for the evaluation of a tumor surrounding the anus (Fig. 2A). Biopsy and histopathological analysis identified the tumor as a perianal gland adenocarcinoma. The risk of recurrence and the treatment options were described to the owners, which included surgery, radiation therapy and chemotherapy. Complete surgical excision was considered to be too difficult, as the tumor border was unclear. HTH experimental therapy was recommended and the animal was enrolled in the clinical trial, with the owners’ written informed consent. On day 0, HTH therapy was performed under general anesthesia, which was administered by inhalation of isoflurane. Five needles of the device were inserted into the tumor at 1-cm intervals, and HTH was performed for 10 min at 65°C (Fig. 2B) and repeated one additional time. On day 21, the tumor volume had decreased from that which was observed on day 0 (Fig. 2C). HTH therapy was repeated using the same protocol, however, the dog succumbed one week later due to old age.

### Case 3

**Figure 3:**
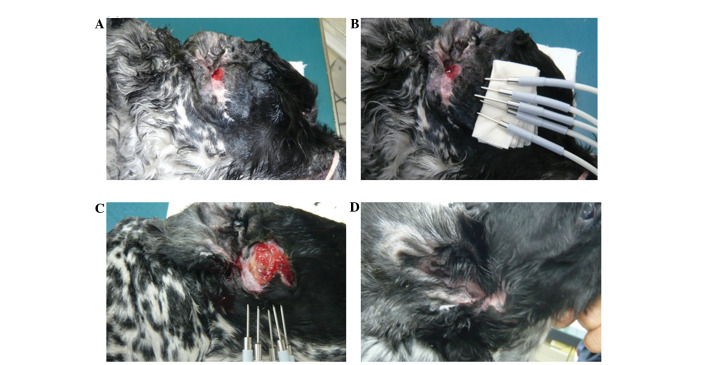
Gross tumor appearance in case 3. (A) The tumor, diagnosed as a ceruminous adenocarcinoma, recurred in the right external auditory canal following a total ear canal ablation. (B) High temperature hyperthermia (HTH) treatment was performed with inhalation of isoflurane. Five needles of the ablation device were inserted into the tumor, and HTH treatment was performed for 10 min at 65°C. (C) The tumor volume decreased and HTH treatment was repeated, using the same protocol, on day 28. (D) The gross appearance of the affected ear on day 133 demonstrates that the tumor disappeared.

A 13-year-old male English Cocker Spaniel (weight, 12.3 kg) was referred to the Takagi Animal Clinic in February, 2011 for the evaluation of a tumor in the right external auditory canal (Fig. 3A). A right total ear canal ablation was performed and subsequent histopathological analysis revealed a ceruminous adenocarcinoma. Two months after the intervention, the tumor recurred at the surgical site. The risk of recurrence and the treatment options were explained to the owner, which included surgery, radiation therapy and chemotherapy. Specifically, surgery presented the risks of vestibular disorders and facial paralysis. HTH experimental therapy was recommended and the animal was enrolled in a clinical trial, with the owner’s written informed consent. On day 0, HTH therapy was performed under general anesthesia, which was maintained using inhaled isoflurane. Five needles of the device were inserted into the tumor and HTH therapy was performed for 10 min at 65°C (Fig. 3B). On day 22, the tumor volume had decreased from that which was observed on day 0. On day 28, the HTH therapy was repeated using the same protocol. On day 78, the tumor volume had decreased further and a third HTH procedure was performed. On day 133, the tumor had disappeared and did not recur.

## Discussion

To the best of our knowledge, the beneficial effects of HTH therapy for the treatment of superficial tumors have not yet been reported in veterinary medicine. The HTH protocol used in the current study was simple to conduct and was only performed on spontaneous tumors that had presented in canines. In the three cases presented, the tumor volumes decreased following HTH therapy; furthermore, no severe side effects were observed in any of the cases.

In recent years, various innovative and minimally invasive cancer therapies have been developed as alternatives to surgery. Ablation, which uses high temperatures, radio waves or microwaves, is considered to be a potent alternative therapeutic strategy (14).

High temperatures (>46°C) directly damage cells, resulting in severe protein denaturation and DNA damage (15,16), which induces irreversible changes that ultimately result in cell death. Tumor cells express specific tumor-associated antigens and in high temperature conditions (>46°C), the tumor cells swell and break into fragments, which releases antigens; this large antigen load generates antitumor immunity. The high temperatures also lead to severe protein denaturation that appears to destroy the immunogenicity of tumor cells (17–21). When thermal ablation temperatures (>70°C) are achieved, there is a high risk of shock syndrome that is induced by the sudden and large production of necrotic tumor material (22). Therefore, the case for ablation therapy in medicine is limited. Ablation therapy is commonly performed on tumors measuring ≤3 cm in diameter (23). In the present cases, tumor sizes were >3 cm in diameter, although this was not measured precisely. In our previous study, it was reported that HTH therapy administered at temperatures between 50 and 70°C induces necrosis and apoptosis in a rat glioma model (13). However, HTH therapy at 50°C did not exert adequate suppressive effects when compared with treatment at 60 and 70°C. The present results coincide with our previous data. The optimal therapeutic protocol, including the effective temperature, time and frequency must be established in order to extend the application of HTH therapy for routine use in veterinary oncology.

In conclusion, HTH treatment is a simple therapeutic option with no severe side effects and is expected to become a useful alternative therapy for superficial tumors in companion animals.
